# Nucleus Accumbens: A Therapeutic Target for Radiomodulation for the Treatment of Alcohol Use Disorder

**DOI:** 10.7759/cureus.88314

**Published:** 2025-07-19

**Authors:** Roshan Amurthur, Neelan J Marianayagam, David J Park, Steven D Chang, M. Bret Schneider, John Adler

**Affiliations:** 1 Science, The Harker School, San Jose, USA; 2 Neurosurgery, Stanford University School of Medicine, Palo Alto, USA; 3 Radiation Oncology, Zap Surgical Systems, Inc., San Carlos, USA; 4 Psychiatry, Stanford University School of Medicine, Palo Alto, USA

**Keywords:** addiction, alcohol use disorder, neuromodulation, nucleus accumbens, radiomodulation

## Abstract

Despite progress in pharmaceutical and behavioral treatments, alcohol use disorder (AUD) continues to have a high relapse rate. While deep brain stimulation (DBS) of the nucleus accumbens (NAc) has shown therapeutic promise, its invasive nature poses significant risks. Radiomodulation, the focal delivery of sub-ablative ionizing radiation, offers a non-invasive alternative for neuromodulating deep brain structures. However, optimal targeting, dosing, and rationale for radiomodulation in AUD have not been discussed. This paper analyzes the NAc as a candidate target for radiomodulation and proposes NAc radiomodulation as a novel, potentially transformative therapeutic approach for AUD that warrants further investigation.

## Introduction and background

Alcohol use disorder (AUD) is defined by the American Psychological Association’s Diagnostic and Statistical Manual of Mental Disorders, Fifth Edition (DSM-5) as a series of self-destructive physical and behavioral symptoms surrounding alcohol use, including withdrawal, tolerance, and craving [1]. AUD is closely related to addiction, which has been described as positive enforcement-driven impulsivity, eventually escalating to negative enforcement-driven compulsivity [2]. While not all persons with AUD are addicted, a substantial portion likely meet the generally accepted definition of addiction [3]. Despite advancements in behavioral and pharmacological treatments, more than 46% of patients with AUD who seek treatment relapse within just one year [4]. The neurochemical profile of AUD is well categorized. Acute alcohol consumption enhances inhibitory, GABAergic activity while suppressing excitatory, glutamatergic signaling in the reward system, resulting in euphoric sensations, sedation, disinhibition, and anxiolysis. This inhibitory state is transient and reverts once alcohol is metabolized. However, with prolonged use, the brain establishes a new homeostatic set point, elevating baseline GABAergic tone. This adaptation motivates increased, toxic levels of alcohol intake to achieve the same euphoric states. This cycle of increased consumption to achieve similar reward states drives dependence and AUD [5].

Functional reward circuitry maintains a stable equilibrium between excitatory and inhibitory neurotransmission. When this balance chronically shifts in one direction, homeostatic mechanisms activate to restore it to equilibrium [6]. To counterbalance chronic alcohol-associated inhibition and maintain homeostasis, the brain lowers GABAergic tone overall and increases glutamatergic signaling via upregulating excitatory N-methyl-D-aspartate (NMDA) receptor expression [7]. Thus, without alcohol, the system becomes hyperexcitable since there is a large imbalance favoring glutamatergic signaling. This culminates in withdrawal symptoms like cravings, seizures, and anxiety. Even when the patient is not actively intoxicated but chronically drinking, the ability to modulate normal emotional regulation, stress response, and cognitive function is compromised because the neurotransmitter system is imbalanced [5]. Thus, neuromodulatory approaches must consider the unique profile of neurotransmission in AUD and the eventual dysregulated state of the brain due to increased adaptation.

“Radiomodulation” is an emerging concept for administering precisely targeted external ionizing radiation (stereotactic radiosurgery) as a clinical procedure for altering the function of brain networks without ablation [8]. Ablation, in contrast, involves the destruction (lesioning) of affected neural tissue, typically through surgical removal, radiofrequency, high-intensity focused ultrasound (HIFU), or the application of high-energy ionizing radiation to permanently disrupt function [9]. Given the combination of high spatial fidelity and non-invasiveness, radiomodulation may have the potential to treat AUD without the surgical risks associated with more invasive deep brain stimulation (DBS). In contrast to other theoretical non-invasive neuromodulatory treatments for AUD, such as transcranial magnetic stimulation (TMS), transcranial direct current stimulation (TDCS), and optogenetic stimulation, the effect of radiomodulation could also potentially be permanent (or nearly so) after a single session. Although not yet proven, radiomodulation may be capable of inducing circuit-wide neuromodulatory effects beyond a focally treated target. While radiomodulation may be effective for other addictive disorders, this paper proposes the application to AUD due to its unique neural circuitry and clinical profile.

Within the domain of AUD therapies, the nucleus accumbens (NAc) has been gaining traction as a logical target for neuromodulation. In this paper, we discuss the feasibility and rationale for utilizing radiomodulation to target the NAc in AUD. We include a narrative review of the anatomy, physiology, and structural connections of the NAc as well as previous work on using DBS to treat AUD. Ultimately, we seek to introduce a novel paradigm for using radiomodulation to treat the NAc in AUD patients.

## Review

Nucleus accumbens: Structure and function in AUD

*Functional Profile in AUD*

**Figure 1:**
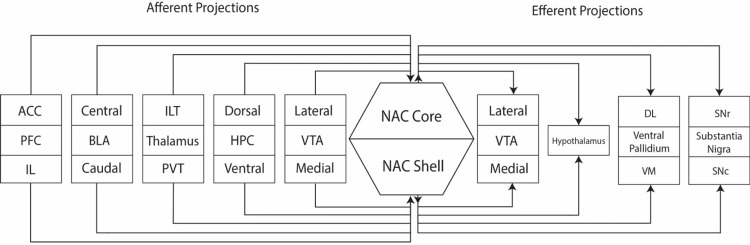
Circuitry of the nucleus accumbens. VTA: Ventral tegmental area; HPC: hippocampus; ILT: inferior lateral thalamus; PVT: posterior ventral thalamus; BLA: basolateral amygdala; PFC: prefrontal cortex; ACC: anterior cingulate cortex; IL: infralimbic cortex; DL: dorsolateral; VM: ventromedial; SNr: substantia nigra pars reticulata; SNc: substantia nigra pars compacta.

The NAc has long been known to be part of the “reward system” of the brain, playing a key role in behavioral reinforcement [10]. The connections making up the NAc circuitry are complex and varied (Figure 1) [11-13]. As such, the NAc is the mesolimbic dopamine system’s primary target, with axonal projections stemming from neuronal bodies in the ventral tegmental area (VTA) [14]. In this manner, it serves as a limbic-motor interface, wherein learned motivation is converted into goal-driven action. Cue-induced firing of NAc neurons promotes reward-seeking behavior [15]. Dopamine levels in the NAc increase in anticipation of reward [16]. In the context of AUD, anticipatory dopamine release likely augments craving and drives the patient toward relapse. Neurochemical studies of the NAc find high extracellular dopamine levels correlated with a stimulating sensation [17]. These factors suggest that AUD is driven by reduced baseline NAc dopamine and hyperexcitation of the dopaminergic system in response to alcohol-craving cues [18]. In the setting of addiction, a therapeutic benefit from neuromodulation may result from resetting baseline dopamine deficits within the NAc.

*Structural Composition*

**Figure 2:**
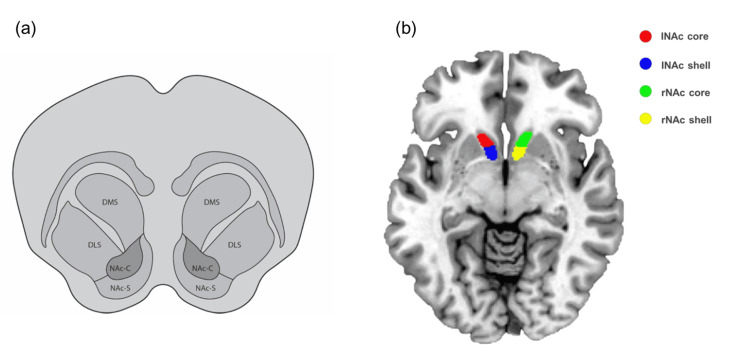
(a) Close-up diagram of the structural composition of striatum. (b) Coronal section of human nucleus accumbens (NAc). DMS: dorsomedial striatum; DLS: dorsolateral striatum; NAc-C: nucleus accumbens core; NAc-S: nucleus accumbens shell. Image (b) is adapted from Wang et al. (2019) [25] under the Creative Commons Attribution 4.0 International License.

Magnetic resonance imaging (MRI) estimates the volume of the NAc to be 473.3 mm^3^ (SD = ±106.8), and can be divided roughly into a core and a shell, each with separate external circuit connections to other brain regions (Figure 2) [19,20]. Dopaminergic pathways indicate functional differences between the shell and core. To date, however, there is no consensus as to either region being a preferable neuromodulation target in the setting of addiction and specifically AUD. By virtue of its extensive VTA projections, the NAc shell is more closely connected with the mesolimbic system and appears to be involved in context-induced renewal of drug and alcohol-seeking behavior [11]. Alcohol preferentially activates rostral NAC shell kappa-opioid receptors, which modulate approach/avoidance behavior for AUD stimuli [21]. Alcohol injection of 2-4 mg/kg in rats increases NAc shell dopamine levels and maintains chronic alcohol dependency [22]. These factors indicate that neuromodulation of the NAc shell may be most promising for the treatment of AUD. In contrast, the NAc core is more closely associated with the nigrostriatal system, a pathway responsible for behavior pertaining to locomotion and motivation [23]. NAc shell but not core appears to be responsible for the affective encoding of reward-associated motivational states in rats [24].

Cellular Composition

Medium spiny neurons (MSNs) constitute 95% of NAc cells and project GABAergic outputs to multiple regions (Figure 1) [26]. MSN activity is heavily regulated by dopaminergic inputs from the VTA via dopamine receptor D3R [27]. Pharmacological blockage or genetic knockdown of NAc-D3Rs amplifies MSN-facilitated GABAergic inhibition and reduces voluntary alcohol intake in mice [7]. These findings corroborate predominant models of AUD, in which elevated glutamate levels and GABA deficits during withdrawal drive craving behaviors [5]. Electrophysiological study in mice demonstrates that MSNs in the NAc shell exhibit higher firing rates and intrinsic excitability than in the NAc core [28]. Thus, inducing an action potential in NAc shell MSNs could be feasible at lower levels of neuromodulation. In addition to dopaminergic afferents from the VTA, the NAc receives glutaminergic projections from the prefrontal cortex (PFC), amygdala, hippocampus, and thalamus [5]. Thus, radiomodulation of the NAc as a central hub of addiction could restore function to a wide range of neural circuits.

Preclinical evidence of NAc neuromodulation for AUD

Several preclinical studies of NAc neuromodulation in alcohol-addicted models indicate positive outcome measures, such as acute and chronic reduction in alcohol consumption [29,30]. Morphine conditioned place preference (CPP) is blocked by 130 Hz NAc-DBS in rats [31]. Stimulation was accompanied by a significant decline in VTA and NAc glutamate and an increase in gamma-aminobutyric acid (GABA) levels when compared with sham and control groups. Kuhn and colleagues concur that NAc neuromodulation reduces the synaptic efficiency of the dopaminergic system, thereby silencing excitatory neurotransmission [32]. It is plausible that DBS functions by affecting both neuronal and glial microenvironments [33]. While high-frequency inhibition of neurons may be a local effect, activation of astrocytes due to high-frequency stimulation likely modulates synaptic activity, resulting in a network-wide effect [33].

Clinical evidence of NAc neuromodulation for AUD

Clinical efficacy of DBS for the treatment of AUD has been studied in a series of case reports and smaller pilot studies and is summarized below (Table 1). In general, these studies indicate the attenuation of alcohol-related cravings and, in some instances, have shown long-term abstinence. A more recent, first double-blinded randomized controlled trial of NAc-DBS confirmed abstinence-promoting effects. Although the study failed to meet the primary endpoint due to the small sample size (n = 12), it demonstrated significant improvement in secondary outcomes, including proportion of abstinent days, heavy drinking days, alcohol craving, and anhedonia [34].

**Table 1:** Early clinical efficacy and outcomes of NAc-DBS for AUD. NAc: nucleus accumbens; DBS: deep brain stimulation; SD: standard deviation; AUDIT: Alcohol Use Disorders Identification Test; AUQ: Alcohol Use Questionnaire; NNT: number needed to treat; OCDS: Obsessive Compulsive Drinking Scale. * Manually calculated, no mean or standard deviation specified in the original study. Values by Davidson et al. [35] and Müller et al. [36] are significant at p < 0.05. Studies by Kuhn et al. [32,37] do not report significance (n = 1). Unless otherwise reported, the studies do not formally report effect size.

Study	Sample size	Notable outcome measure	Mean pre-op/control (SD)	Mean post-op/experimental (SD)	Effect size/outcome metric
Bach et al. (2023) [34]	n = 12 (6 DBS-Early on, 6 DBS-Late on)	Abstinent days (% improvement in previous 30 days, secondary outcome)	DBS-Early on: 71.0 (32.9)	DBS-Late on: 40.8 (22.7)	Hazard ratio (HR) = 0.73 (95% CI: 0.20–2.62); NNT = 9.6 (95% CI: 1.9–16.4)
Davidson et al. (2022) [35]	n = 6	AUDIT	Pre-op: 35.7 (3)	Post-op: 12 m: 11.0 (12)	Cohen’s d ≈ 0.7 (alcohol consumption), d ≈ 1.7 (craving/OCDS); large, significant reductions
Müller et al. (2016) [36]	n = 5	AUQ	Pre-op: 30.6 (13.66)*	Post-op: 8 (0)*	Complete absence of craving in all; 2 long-term abstinent, 3 marked reduction
Kuhn et al. (2011) [32]	n = 1	AUDIT	Pre-op: 32	Post-op: 12 m: 14	No standardized metric; marked reduction in consumption with DBS
Kuhn et al. (2007) [37]	n = 1	AUDIT	Pre-op: 28	Post-op: 1	No formal effect size; marked reduction in daily drinks and urge post-DBS

Some studies do not disclose a specific subregion. Moreover, other reports could be misleading were an inaccurate DBS procedure misses its proper target [32]. Accurate placement of electrodes has been shown to be the difference between abstinence and relapse [38]. Finally, increases in voltage due to glial scarring can be a confounding variable that results in current spread from electrodes, thereby subtracting from apparent DBS precision and contributing to misreported target locations. Despite promising results, current DBS studies rarely differentiate between stimulation of the NAc core versus shell. Few, if any, studies achieve primary clinical endpoints, leaving a gap in justification for circuit-specific targeting. Effect size and statistical significance were not consistently reported across studies. Thus, comparisons should be interpreted with caution.

DBS safety and limitations

Most adverse events that occur during invasive NAc-DBS trials are primarily related to surgery [39]. Surgical case studies of NAc ablation document patients sustaining injuries, including dysphoria, anterograde amnesia, affective disorders, and motivational dysfunction [10,39]. While potentially a result of direct NAc ablation, it is possible that damage to surrounding structures could also have been a contributing factor. Impairment of the olfactory cortex, which has extensive connections with the NAc, might underlie dysphoria and motivational dysfunction, as well as memory disorders could be a result of damage to the orbitofrontal cortex [40]. Furthermore, stimulation of ventral portions of the NAc can potentially alter mood and exacerbate pre-existing depression, since the NAc is implicated in functions beyond solely motivation [41]. However, a clinical study of NAc/ventral capsule DBS for chronic opioid use reported reductions in depression and anxiety metrics as well as physiological and cognitive functioning [42].

Aside from behavioral considerations associated with targeting deep brain structures, electrode implantation carries direct surgical risks like infection, seizure, intracerebral hemorrhage, and ischemic infarction [43]. Furthermore, hardware malfunctions, lead misplacement, discomfort, and battery damage are long-term complications associated with traditional, invasive equipment [44]. While rare, these surgical and equipment-related risks may be severe. Thus, an alternative to invasive procedures could be beneficial to many patients, especially starting with those who are not good surgical candidates.

Radiomodulation

Radiomodulation is neuromodulation via focally delivered non-lesional ionizing radiation. It is to be differentiated from radiosurgical ablation, which intentionally destroys neural tissue to achieve therapeutic outcomes. Ablation often involves high-dose ionizing radiation that permanently disrupts neural activity by causing cellular death in targeted regions. In contrast, radiomodulation employs “small volume” sub-ablative doses (typically below 40 Gy), which may induce synaptic plasticity and alter neural circuitry without significant structural damage [45]. When delivered on stereotactic radiosurgery platforms, radiomodulation possesses the ability to non-invasively target small volumes with sub-millimetric accuracy [46]. Furthermore, focal low-dose irradiation induces durable plasticity that extends beyond the initial target while preserving the overall function of neural networks.

The radiomodulation effect appears to be bimodal and permanent, achieving durable up-regulation or downregulation of the target depending on the dose delivered [47]. The mechanisms underlying this effect are still the topic of investigation. Multiple candidates are likely involved and include differential biological effects upon specific populations of neurons, glia, and vasculature [45]. In an in vitro induced pluripotent stem cell (iPSC)-derived assay, inhibitory neurons were found to have higher sensitivity to ionizing radiation than their excitatory counterparts [45]. An interesting related observation is that the binding of muscimol (a GABA agonist) decreases following irradiation, supporting the contention that relatively low doses upregulate neural circuits [48].

Radiation doses of 40 Gy and below appear to induce synaptic plasticity that can remodel the activity of downstream neural circuits without significant structural damage [45]. In contrast, focal radiation doses of 60-100 Gy downregulate neural motor circuits in pigs and are associated with partial cell death [49].

Radiomodulation could interact with NAc MSNs at the level of D3R input downregulation and/or GABAergic output upregulation to restore top-down executive control and temper AUD-associated cravings. Neurons exposed to moderate doses of radiation exhibit more than 50% reduction in firing rates sustained over several months. Alternatively, ionizing radiation can induce persistent changes in the sodium channel function, effectively stabilizing neurons in a hyperpolarized state. By making neurons less responsive to depolarizing inputs, radiomodulation can reduce hyperactivity [8]. Therefore, delivering sub-ablative dosing to the NAC shell might reduce excitability of MSNs, effectively dampening the maladaptive loop. Thus, radiomodulation-induced dampening could return local circuits to the normal physiological range of excitability. This shift could then help to restore balance and diminish AUD symptoms.

The choice of dosing parameters and potential adverse effects of radiomodulation will need to be considered when targeting the NAc. Functional radiosurgical procedures in mice showed clinical effects with minimal to no tissue destruction [50]. In irradiated minipigs, the changes induced in glial cells were implicated as a potential mechanism of action, and cellular damage was not observed [51,52]. The selective vulnerability of certain neuronal subtypes may result in astrocytic damage, even at low doses, which leads to alteration of the glial microenvironment [53]. The effect of radiomodulation on glial cells should be investigated further. Furthermore, low-dose radiation may still cause unintended synaptic plasticity in non-target regions. While Zaer and colleagues studied molecular mechanisms in neuron-astrocyte co-cultures, experiments comparing separate in vitro assays of neuron and glial cultures could unlock further insights [45].

Discussion

While still in the domain of research, the NAc is increasingly being investigated as a viable DBS neuromodulatory target for the treatment of AUD [16-20]. While these studies show promising efficacy, invasive stimulation carries the risk of infection, bleeding, and neurological disability. Long-term DBS complications, such as equipment failures and lead misplacement, suggest a need for non-invasive therapies. Furthermore, the NAc is implicated in a range of neurological and psychiatric disorders such as obsessive-compulsive disorder (OCD), anxiety, and depression, making ablation and neuromodulation potentially risky and complex [54]. However, preliminary evidence of the therapeutic effects of NAc-DBS in combination with an improved classification of AUD pathology and NAc structure/function indicates promising potential for radiomodulation.

NAc sub-region selection is important to maximize the efficacy of treatment. While there is no clear consensus regarding the preferred target and further study would be beneficial, the NAc shell is associated with the mesolimbic system, marked by extensive dopaminergic projections to the VTA. As a key player in the limbic system, the shell may mediate emotional stimuli associated with addiction. The NAc core is associated with locomotive and motivation-related behavior, indicating that targeting the NAc core is less likely to durably alter the necessary circuits in AUD to achieve therapeutic outcomes. Furthermore, since NAc shell MSNs display higher baseline excitability, lower doses of radiation could be sufficient to induce plastic changes. Thus, NAc shell irradiation may be safer, since high-dosage treatments that risk cell death may not be required. Heightened impulsivity in AUD is likely a result of dysregulation of the mesolimbic system and a loss of top-down control from the medial prefrontal cortex (mPFC) [18]. The NAc-shell receives projections originating from cortical areas of the limbic system, such as the mPFC and ventral hippocampus (Figure 1). Dopaminergic VTA projections terminate mainly in the shell. High-frequency stimulation of NAc shell but not core is associated with significant GABA increase and subsequent neuronal firing decrease in the lateral hypothalamic area, a region implicated in orexin-based reward processing [55,56]. NAc core stimulation functions similarly in some preclinical work but does not reduce voluntary alcohol consumption in other studies. Therefore, it appears that NAc shell stimulation may more effectively modulate impulsive behavior by re-establishing normative signaling in mesolimbic circuitry and enhancing top-down cortical regulatory control. Withdrawal in AUD is characterized by heightened glutamate levels and depleted GABA signaling, driving hyperexcited cravings to re-establish baseline GABA and associated euphoric states [5]. In the NAc, dopaminergic input regulates GABAergic MSNs, the primary neuronal population. It is hypothesized that NAc radiomodulation could produce therapeutic effects by resetting dysregulated GABA circuitry and re-establishing baseline GABA levels, preventing craving.

Radiomodulation in pigs with low doses seems to deliver an upregulatory effect to overall brain circuits at 40 Gy or less, potentially due to the differential response of inhibitory neurons [45]. Since AUD withdrawal involves a glutamate-driven state of hyperexcitation, radiomodulation could prove to be a beneficial, non-invasive alternative therapy to DBS for AUD. In the context of AUD, preclinical studies may show D3R dopamine receptor expression and MSN GABAergic activity post irradiation to validate potential mechanisms. Studies aimed at further verifying the molecular mechanisms of radiomodulation are necessary and may provide additional insight into effective dosing and targeting of AUD. While promising, radiomodulation may result in damage to astrocytes and the broader glial microenvironment at even low doses. In addition to dependence on anatomical target, dosing is contingent on individual variability between patients. Future work should focus on dose optimization in primate models. Longitudinal studies of radiomodulation are required to gauge long-term safety as well as verify the efficacy of treatment. Compared to radiomodulation, non-invasive therapies such as TDCS and TMS are relatively non-focal and are mainly capable of targeting superficial brain regions. Thus, radiomodulation uniquely may be posed to deliver non-invasive stimulation with comparatively high fidelity. While promising, radiomodulation remains experimental and requires rigorous safety testing.

## Conclusions

In this paper, we have discussed the NAc as a potential therapeutic target for the treatment of AUD utilizing stereotactic radiosurgery. For patients with severe AUD who are refractory to conservative treatments, DBS targeting the NAc has provided positive clinical results. Clinical study of NAc-DBS shows an increase in abstinent days, reduction of cravings, improved impulse control, and reduced relapse.

However, the potential for adverse consequences, including surgical complications like infection and equipment malfunctions, points to the need for a non-invasive, durable treatment option. Radiomodulation of the NAc shell region may offer a solution for patients with reduced risk of complications, faster recovery time, and more reliable outcomes. Radiomodulation exhibits a bimodal, durable effect, which may alter circuit-wide connections in the NAc to restore dysfunctional AUD circuitry. The radiomodulatory effect occurs at sub-ablative doses of radiation. Preclinical research aimed at evaluating dosage for irradiation of the NAc shell in animal models, investigation of potential mechanisms of action, and validation of safety profiles are all necessary next steps in the process of adopting radiomodulation as a potential therapy for AUD.
